# Rheological and Structural Evaluation of Dental Flowable Composites for Optimized Performance in Transparent Aligner Systems

**DOI:** 10.3390/polym18111308

**Published:** 2026-05-26

**Authors:** Elena Palmieri, Maria Elena Cataldi, Loredana Cerroni, Luca Montaina, Matteo Bonomo, Gaetana Petrone, Denise Bellisario, Leonardo Mattiello, Guido Pasquantonio, Andrea Liscio, Francesco Maita, Luca Maiolo, Roberta Condò

**Affiliations:** 1 Institute for Microelectronics and Microsystems, National Research Council, CNR-IMM, Via del Fosso del Cavaliere, 100, 00133 Rome, Italy; elena.palmieri@cnr.it (E.P.); lucamontaina@cnr.it (L.M.); gaetanapetrone@cnr.it (G.P.); andrea.liscio@cnr.it (A.L.); luca.maiolo@cnr.it (L.M.); 2 Department of Clinical Sciences and Translational Medicine, University of Rome “Tor Vergata”, Via Montpellier, 1, 00133 Rome, Italy; cataldi.doc.elena@gmail.com (M.E.C.); cerroni@uniroma2.it (L.C.); guido.pasquantonio@uniroma2.it (G.P.); 3 Department of Basic and Applied Science for Engineering, SBAI Sapienza, University of Rome, Via del Castro Laurenziano, 7, 00161 Rome, Italy; matteo.bonomo@uniroma1.it (M.B.); leonardo.mattiello@uniroma1.it (L.M.); 4 Research Center for Nanotechnologies Applied to Engineering, CNIS Sapienza, University of Rome, Piazzale Aldo Moro, 5, 00161 Rome, Italy; 5 Department of Electrical and Energy Engineering, DIEE Sapienza, University of Rome, Via Eudossiana, 18, 00184 Rome, Italy; 6 Department of Industrial Engineering, University of Rome “Tor Vergata”, Via del Politecnico, 1, 00133 Rome, Italy; denise.bellisario@uniroma2.it

**Keywords:** flowable resin composites, polymeric dental materials, rheological behavior, filler–matrix interaction

## Abstract

Clear aligner therapy (CAT) increasingly relies on composite-based attachments to improve force transmission and aligner retention, yet the role of flowable composite properties in clinical performance remains poorly understood. In this study, five commercially available flowable composites used for orthodontic attachments—Aligner FLOW LC, SIMPLY SHADE, SOFT ENA Flow, TETRIC EvoFlow, and VENUS Bulk Flow One—were comparatively investigated through physicochemical, morphological, optical, thermal, and rheological characterization. Scanning electron microscopy coupled with energy-dispersive X-ray analysis, thermogravimetric analysis, UV–Vis–NIR and ATR–FTIR spectroscopy, and rheological measurements before and after curing were employed to probe composition, filler content, viscoelastic behavior, and mechanical response. The results revealed marked differences among the investigated materials, with post-curing storage modulus spanning nearly two orders of magnitude, from 0.06 MPa for SOFT ENA Flow to approximately 5 MPa for SIMPLY SHADE. Similarly, the elastic modulus ranged from about 20 MPa to nearly 1000 MPa for the softest and stiffest resins, respectively. Interestingly, SOFT ENA Flow, the softest material after curing, also exhibited the highest pre-curing viscosity, nearly one order of magnitude greater than the least viscous resin, Aligner FLOW LC. These findings highlight an intrinsic trade-off between pre-cure processability and post-cure mechanical stability, providing a rational framework for material selection in orthodontic attachments and supporting more predictable and durable CAT outcomes.

## 1. Introduction

The landscape of contemporary orthodontics has been profoundly reshaped by the emergence of Clear Aligner Therapy (CAT), a revolutionary approach employing a sequential series of nearly invisible, removable thermoplastic aligners [1]. Despite the limitations of clear aligner therapy (CAT), particularly in the predictability of complex orthodontic movements such as rotations, extrusion, and root control, as well as its strong dependence on patient compliance and on the biomechanical performance of aligners and attachments, this innovative method has become increasingly widespread. Indeed, CAT offers a highly esthetic and comfortable alternative to traditional fixed orthodontic appliances, rapidly gaining popularity and fundamentally altering patient expectations and treatment paradigms. CAT facilitates controlled tooth movements by guiding teeth into pre-planned positions through incremental adjustments [2]. Despite the inherent esthetic and comfort advantages of clear aligners, their clinical efficacy is inextricably linked to the precise design and proper application of attachments. These small, auxiliary structures, typically fabricated from an esthetic composite material, are adhesively bonded to the enamel surface of teeth. Their critical role lies in serving as essential anchorage points and effective systems for transmitting the intricate orthodontic forces generated by the aligners. The specific form, dimension, and precise position of these attachments are crucial for achieving optimal three-dimensional control over complex dental movements, such as rotations, extrusions, or translations, thereby optimizing the critical biomechanical interaction between the aligner and the tooth [3]. Attachments provide supplementary force vectors, transforming the force system to enable movements otherwise difficult to achieve with aligners alone, and have been shown to significantly increase the efficiency and predictability of specific tooth movements, including torque, expansion, rotation, intrusion, and translation. Studies confirm that load transfer from aligners to teeth is notably limited without attachments, underscoring their indispensable role in achieving desired dental movements. Their design, encompassing geometry, position, and dimension, is therefore a fundamental determinant of treatment success. Key functions include providing enhanced aligner retention, preventing unwanted aligner slippage on rounded tooth surfaces, and delivering precise, predetermined force vectors [4]. The materials predominantly used for fabricating these orthodontic attachments are resin composites. These are multiphase materials, generally composed of a continuous organic resin matrix and dispersed inorganic reinforcing fillers, combined to yield superior mechanical and physical properties than their individual constituents [5,6]. While various types of composites exist for this purpose, flowable composites are frequently favored due to their inherent fluidity, which greatly simplifies their application and adaptation into the precise, predefined geometries of attachment templates [7]. Flowable composites are characterized by a lower viscosity, owing to a reduced filler content (typically 37–70% by volume) and a modified resin composition. This low viscosity allows them to flow effortlessly and adapt accurately to the complex morphologies of aligner templates, including undercuts and hard-to-reach areas, ensuring a precise replication of the attachment form [4]. Ideal composites for attachments must exhibit adequate hardness and wear resistance to maintain their shape throughout treatment, strong adhesion to enamel, sufficient viscosity and moldability for precise reproduction, controlled opacity for clinical visibility without compromising esthetics, and long-term dimensional and chromatic stability in the oral environment [8,9]. However, the selection of the most appropriate composite material for attachments is a critical consideration for ensuring clinical efficacy and long-term stability [5,10,11,12]. The rheological properties of flowable composites play a key role in their clinical performance, as they determine both handling during application and the mechanical behavior after curing. In particular, parameters such as viscosity and viscoelastic moduli influence the ability of the material to properly fill the aligner template in the uncured state and to retain shape and transmit forces once polymerized. While low viscosity facilitates adaptation and template filling, excessively fluid systems may lead to uncontrolled spreading before curing; conversely, higher stiffness after curing is required to ensure dimensional stability and effective force transfer [12,13,14,15,16,17].

The selection of the appropriate composite is therefore a critical step in attachment fabrication, as the material must combine adequate processability before curing with sufficient mechanical stability after polymerization. Despite their widespread clinical use, a substantial gap remains in the scientific literature regarding the rheological, chemical, and mechanical properties of flowable composites used for orthodontic aligners. Although some recent studies have begun to explore this topic [18,19], the vast and ever-expanding variety of resins, constantly updated to meet changing clinical needs and advances in aligner technology, makes it difficult to establish a comprehensive understanding. Additional information and a more detailed discussion of the clinical aspects are provided in the Supporting Information SI (Section: “Rationale for Attachments in Aligner Therapy”, Figure S1).

In this context, the aim of this work is to establish a direct relationship between the physicochemical and rheological properties of flowable composites before curing and their mechanical and structural behavior after polymerization. Five commercially available materials commonly used for orthodontic attachments (Aligner FLOW LC, SIMPLY SHADE, SOFT ENA Flow, TETRIC EvoFlow, and VENUS Bulk Flow One) were analyzed through a combination of rheological, thermal, spectroscopic, and morphological techniques.

The goal is to provide a material-based framework to support clinicians in selecting the most appropriate composite for attachment fabrication by balancing handling characteristics with post-curing mechanical performance. At the same time, this study demonstrates how filler content, matrix chemistry, and matrix–filler interactions collectively govern the relationship between uncured processability and post-cured mechanical stability, ultimately influencing the predictability of aligner treatment.

## 2. Materials and Methods

### 2.1. Materials

**Figure 1:**
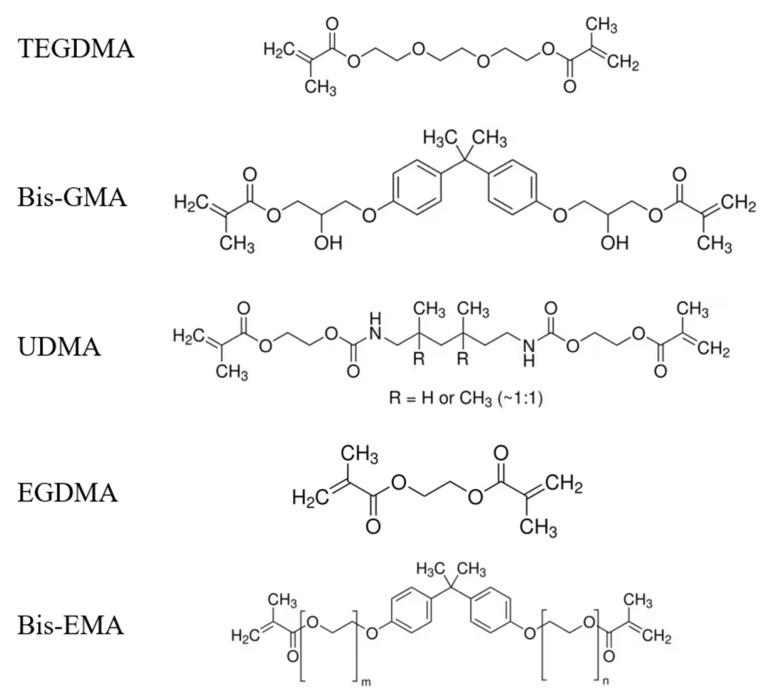
Structural formulas of methacrylate-derivative monomers commonly used for dental applications.

The flowable resins tested are mainly composed of polymers belonging to the dimethacrylate (DMA) family, more specifically HEDMA (2-hydroxyethyldimethacrylate), Bis-GMA (bisphenol A-glycidyl methacrylate), TEGDMA (triethylene glycol dimethacrylate), Bis-EMA (bisphenol A diglycidyl methacrylate ethoxylated), EGBADMA (ethylene glycol bisphenol A dimethacrylate), UDMA (urethane dimethacrylate), and DUDMA (diurethane dimethacrylate). The chemical structures of the most commonly used monomers are reported in Figure 1.

Five commercial flowable resin composites were selected for evaluation. These materials were chosen due to their clinical use as attachments in clear aligner therapy. The composition is generically specified by the manufacturer and reported in Table 1.

**Figure 2:**
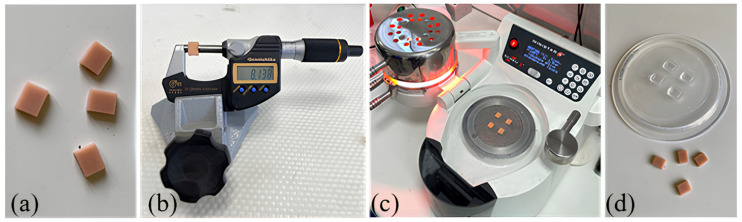
Production workflow for flowable composite resin specimens, (**a**) starting with the creation of an ethyl vinyl acetate mold, achieved after 3D printing four high-precision resin models. The four models were first measured with a micrometer (**b**), positioned at the center of a thermoforming machine plate (**c**), and then covered with an ethyl vinyl acetate disk. Heating the disk produced a negative mold used as a template for fabricating the specimens (**d**). This procedure was repeated five times, thus obtaining five different molds from which samples were realized, three for each of the different flowable composite resins examined.

According to the manufacturers, their filler content ranges from approximately 40% to 80% by weight (pre-curing), with inorganic phases including barium glass, silica, TiO_2_, YbF_3_, initiators and pigments, dispersed in a methacrylate-based resin matrix. For brevity, the samples will from now on be named Aligner FLOW, SOFT ENA, SIMPLY SHADE, TETRIC and VENUS (Figure 2).

### 2.2. Sample Preparation

Four high-precision resin models (PRECISA RD097, Vicenza, Italy) with dimensions of 10 mm × 8 mm × 4 mm were made by a DIGITALWAX 020D 3D printer (DWS s.r.l., Vicenza, Italy) (Figure 2a). Dimensional consistency of the printed models before mold fabrication was verified with a QuantuMike IP65 digital micrometer (Mitutoyo Italiana s.r.l., Milano, Italy) (Figure 2b). The obtained models were used as a reference for the creation of four negative cavities, or templates, positioned at the center of each of the five molds created by vacuum thermoforming with the MINISTAR S thermoforming machine (SCHEU-DENTAL GmbH, Iserlohn, Germany), from the same number of ethylene vinyl acetate (EVA) sheets (Erkoflex, Erkodent Erich Kopp GmbH, Pfalzgrafenweiler, Germany) (Figure 2c). The resulting molds were stored at room temperature in a protected place until the flowable composite resin samples preparation phase (Figure 2d). Each flowable composite resin was handled according to the manufacturer’s instructions, using a manual injection technique with a dispensing syringe provided by the manufacturer and containing the composite resin. The material was inserted into the mold cavity in a standardized single-step procedure for all investigated materials. Once all four cavities were filled, the all surface was then compressed with a 1 mm thick glass plate. Polymerization was performed using a PowerCure Bluephase curing LED light (Ivoclar Vivadent AG, Liechtenstein, Austria), with an emission spectrum of 350–515 nm, as verified with a Demetron Research Corp. radiometer (Demetron Research Corp., Danbury, CT, USA). Exposure times were applied according to the manufacturers’ recommendations for each material (Table 2).

The light was applied to both surfaces (upper and lower), keeping the tip of the light 1 mm from the sample surface. Fifteen rectangular-parallelepiped-shaped specimens (10 mm × 4 mm × 8 mm, Figure 2a,d) were made (*n* = 3 for each resin), using the standardized silicone molds. A standardized specimen thickness of 4 mm was adopted to ensure direct comparison under identical experimental conditions. Although not all investigated composites are specifically indicated for bulk-fill polymerization at this thickness, the selected geometry was intended to provide methodological consistency for the comparative physicochemical and mechanical characterization performed in this study. All specimens were stored at 23 ± 2 °C and 50 ± 5% relative humidity for 24 h before testing.

### 2.3. Morphological and Compositional Characterization of Cured Resins

The surface and fracture morphology of selected specimens were observed under scanning electron microscopy (SEM, Zeiss Sigma 300, Jena, Germany equipped with an EDX probe) after sputter coating with chromium to make them conductive. SEM was used to evaluate filler dispersion, fractured patterns, and surface homogeneity. An EDX investigation was also carried out to confirm the inorganic fraction reported by the manufacturers. No polishing protocols were performed on the examined materials in order to maintain their specific features.

### 2.4. Thermogravimetric Analysis (TGA) on Cured Resins

The mass loss profile (TGA) and its derivative (DTG) were recorded to evaluate thermal stability and inorganic filler content post-curing. Thermogravimetric analyses (TGA) were performed using a TGA 55 thermal analyzer (TA Instruments, Waters Corporation, New Castle, DE, USA). Approximately 10 mg of each resin sample was placed in a platinum crucible and heated under a nitrogen atmosphere from 30 °C to 1000 °C at a heating rate of 10 °C/min. The small amount of material required for TGA measurements was obtained from the cured resin specimens prepared as described in Section 2.2. More specifically, three specimens were fabricated for each resin, and a small portion was cut from each specimen for thermal analysis, resulting in three independent TGA measurements for each material. The instrument was calibrated using standard reference materials. The derivative thermogravimetric (DTG) curves were obtained from the first derivative of the recorded weight-loss data to better resolve overlapping decomposition steps.

### 2.5. UV-Vis and FT-IR Spectroscopy on Cured Resins

To assess translucency and to gain information regarding the composition and the roughness of the attachments, UV–visible reflectance spectra were recorded. The measurements were performed using an Agilent Cary 5000 spectrophotometer (Santa Clara, CA, USA) equipped with an integrating sphere, covering the 200–1500 nm spectral range.

FTIR-ATR characterization was performed using a ThermoScientific Nicolet Summit spectrometer (Waltham, MA, USA) in absorbance mode. Samples were measured in attenuated total reflectance mode (ATR), with a diamond crystal with a single reflection accessory. The spectra were collected from 4000 to 500 cm^−1^ with a resolution of 2 cm^−1^.

### 2.6. Rheological Characterization on Uncured and Cured Resins

Rheological characterization was performed using an HR 20 rheometer by TA Instruments, Waters Corporation, New Castle, DE, USA. As for shear flow measurements, a parallel plate geometry (steel, 20 mm diameter) was used for all the characterizations. The viscosity–shear rate profiles of the various dispersions studied, referred to as ‘flow curves’, were determined at 37 °C in a range of shear rates from 0.01 to 1000 s^−1^. Flow curve graphs (for uncured resins) are presented as double-logarithmic plots of viscosity (Pa·s) vs. shear rate (s^−1^). The linear viscoelastic region (LVR) was determined through preliminary amplitude sweep tests performed at a fixed frequency of 1 Hz, ensuring that subsequent frequency sweep measurements were conducted within the linear regime. Rheological oscillatory characterization was carried out on both uncured and cured samples. For uncured resins, a strain of 0.1% was applied, while for cured samples, a strain of 0.01% was used under a constant normal force of 10 N. The applied normal force ensured proper contact between the sample and the measuring plates, minimizing slip effects. This value was maintained for all samples to guarantee comparable testing conditions, despite slight variations in sample thickness on the order of a few micrometers. Moreover, a custom-made 3D printed sample holder was fixed to the bottom plate to keep the specimen in place during measurements. All experiments were repeated a minimum of 3 times. Errors are not reported in the graphs presented for clarity, and they do not affect the hierarchy of the displayed parameters.

### 2.7. Indentation Testing on Cured Resins

To assess the surface mechanical behavior of the cured composites, flat-ended instrumented indentation tests were carried out using a universal testing machine (Insight/5, MTS Systems S.r.l., Strada Pianezza 289, Torino, Italy). The setup included a flat tungsten carbide indenter with a diameter of 1 mm and a 5 kN load cell. Two specimens for each material type were tested, with each sample securely mounted on a ceramic holder and indented on its flat upper surface.

**Figure 3:**
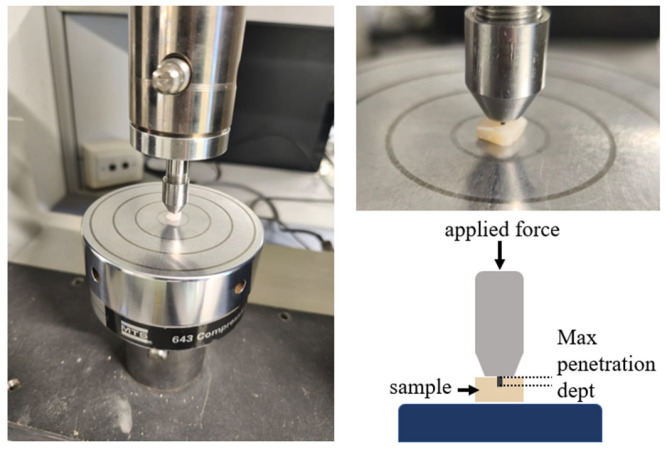
Picture of the flat indentation test setup and schematic representation.

The tests were conducted under displacement-controlled conditions. A preload of 0.5 N was first applied to ensure proper contact, followed by a loading phase at a constant rate of 0.1 mm/min until a maximum penetration depth of 0.25 mm was reached. This depth was selected to remain within approximately 10% of the sample thickness, thereby minimizing the influence of the underlying substrate. Once the target displacement was achieved, the indenter was unloaded back to its initial position. The resulting force–displacement curves were then analyzed to determine mechanical parameters such as stiffness and hardness (see Figure 3).

## 3. Results

### 3.1. Morphology and Elemental Analysis of the Cured Resin

**Figure 4:**
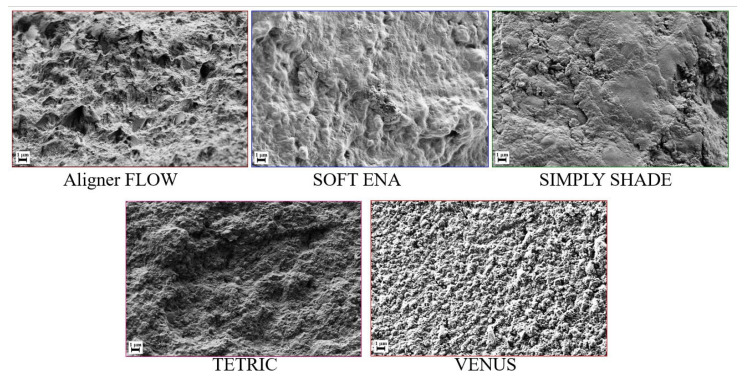
SEM micrographs of the different samples, acquired at 10 kX magnification and an accelerating voltage of 3 keV.

All five samples exhibit broadly comparable surface morphologies, characterized by textured polymeric matrices with inorganic fillers generally well dispersed throughout the material (Figure 4 and Figure S2). The elemental composition was further investigated by EDX analysis, while the Cr signal detected in all samples originates from the metallization process used to improve conductivity during SEM observations.

Among the investigated materials, Aligner FLOW LC displays the most heterogeneous morphology, with micro- and nano-structured features embedded within a highly cross-linked matrix (Figure 4 and Figure S2). EDX analysis confirms the presence of Si, Al, and Ba associated with inorganic fillers (such as silica, barium aluminum borosilicate glass), homogeneously distributed without evident clustering (Figure S3).

SOFT ENA Flow presents a slightly more nanostructured surface, suggesting a somewhat less homogeneous filler dispersion, although no large aggregates are observed (Figure 4 and Figure S2). EDX analysis confirms the presence of Si together with Ti and Al (Figure S4) associated with silica, barium aluminum borosilicate glass, and titanium dioxide.

SIMPLY SHADE exhibits a more irregular morphology composed of central structures surrounded by nanoscale clusters (Figure 4 and Figure S2). EDX mapping indicates homogeneous elemental distribution and reveals traces of Zr, likely associated with impurities in the ytterbium trifluoride filler (Figure S4). Interestingly, no Ba signal was detected despite its declaration in the technical datasheet, possibly due to localized clustering within the matrix.

TETRIC EvoFlow shows a highly homogeneous surface morphology consistent with the coexistence of inorganic fillers and organic copolymers (Figure 4 and Figure S2). Slightly protruding micrometric structures enriched in Si, Al, and Ba are attributable to barium glass fillers, while the presence of YbF_3_ is supported by the overlapping Yb and F elemental maps (Figure S5). Small traces of Zr are again detected.

Finally, VENUS Bulk Flow One displays the most distinctive morphology, characterized by sub-micrometric “cornflake-like” structures uniformly distributed over the surface (Figure 4 and Figure S2). EDX mapping associates these features with YbF_3_-rich regions and confirms the additional presence of Ba-, Al-, and Si-containing glass fillers (Figure S7). Despite the heterogeneous nanostructures, the polymeric matrix remains compositionally homogeneous, as indicated by the uniform carbon distribution.

### 3.2. Thermogravimetric Analysis (TGA and DTG) of the Cured Resin

**Figure 5:**
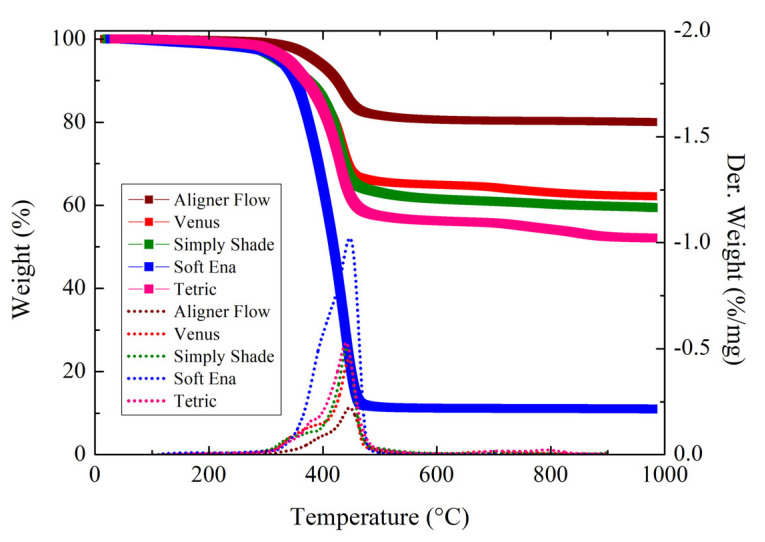
TGA (solid lines) and DTG (dotted lines) curves from 50 °C to 1000 °C for the five resins tested.

The resins examined in this work are primarily based on methacrylate derivatives and consist of a complex blend of organic polymeric components combined with a significant fraction of inorganic fillers. Owing to this multiphase composition, thermogravimetric analysis (TGA) was performed to assess the thermal stability of the polymeric matrix and to provide an approximate estimation of the inorganic filler content from the residual mass after thermal degradation. It should be noted, however, that under inert conditions a minor contribution from carbonaceous char cannot be completely excluded, although methacrylate-based systems typically undergo extensive degradation by 800–1000 °C. The results of the TGA are shown in Figure 5, and the main observations for each resin are discussed below. Aligner FLOW (dark red curve) displayed thermal stability up to about 350 °C, followed by a principal weight loss between 350 and 450 °C, typical of Bis-GMA/TEGDMA-type resin matrices. Its high residual mass (∼80%) confirmed a significant inorganic filler phase consistent with barium glass and silica. SOFT ENA (blue curve) exhibited the lowest residual mass (≈11%), suggesting a more organic-rich formulation. The main decomposition step occurred between 380 and 420 °C; however, the DTG profile shows a complex pattern with broad, overlapping, and poorly resolved shoulders extending from approximately 340 to 380 °C. This behavior is indicative of a multi-step degradation process, likely arising from the presence of organic fillers whose thermal degradation occurs at temperatures comparable to that of the resin matrix. SIMPLY SHADE (olive curve) showed minimal mass loss below 350 °C, with a high final residue (around 61%), roughly matching its manufacturer-declared 75–81 wt% filler. The DTG curve displayed a main degradation peak near 400 °C and minor shoulders at 280 and 330 °C, possibly linked to incomplete polymer crosslinking or additives. TETRIC (pink curve) exhibited a final residue of ~50%, in reasonable agreement with its reported filler content (65–70 wt%). Its degradation occurs mainly between 370 and 450 °C, with a sharp DTG peak at ~400 °C and a minor shoulder around 360 °C, likely associated with partial side-chain decomposition.

VENUS (red curve), despite a declared filler content of 41 wt%, shows a significantly higher residual mass (~60 wt%). While this discrepancy may partly arise from the formation of thermally stable species during degradation, the overall behavior suggests that the residue is largely dominated by the inorganic fraction, indicating a higher effective filler content than nominally reported. The DTG profile of VENUS displays a main decomposition peak at ~400 °C, with a shoulder near 350 °C, indicative of the early degradation of less crosslinked domains.

More generally, DTG shoulders in the range 320–370 °C are observed across all materials and are typically attributed to the degradation of unreacted monomers, additives, or early fragmentation of pendant groups preceding complete network breakdown, which is characteristic of multi-component dental composites [8,11,20]. Additionally, both VENUS and TETRIC exhibit a small but noticeable mass loss above ~600 °C, with a further minor step around ~800 °C. This behavior may be related to the decomposition of thermally stable species, such as carbonates. Accordingly, the residual mass measured at 1000 °C can be reasonably considered to arise predominantly from the inorganic fraction.

In addition to the main degradation process occurring at higher temperatures, all the investigated resins exhibit only a very limited mass loss at low temperatures, attributable to residual moisture or volatile species. Overall, this initial weight loss is negligible for all samples, indicating low moisture uptake. Among the investigated materials, SOFT ENA shows a ‘steeper mass’ loss in the low-temperature region around 100 °C; however, this loss remains very modest (about 2%). For all other resins, the mass loss observed up to 300 °C does not exceed approximately 5%. Importantly, the samples reach comparable residual masses prior to the onset of the main thermal degradation, suggesting a similar low-temperature thermal stability and limited volatile content across the investigated resin systems.

### 3.3. Spectroscopic Characterization of the Cured Resin

**Figure 6:**
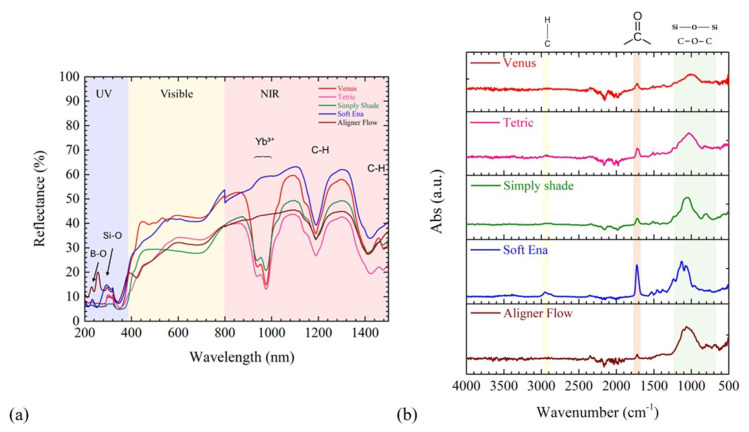
(**a**) Total hemispherical reflectance spectra of the analyzed resin samples; (**b**) ATR-FTIR spectra of the analyzed resin samples.

UV–Vis–NIR spectrophotometry was used to investigate the filler fraction and type of their optical behavior. The samples mainly varied in the type of inorganic phase (ranging from silica alone to more complex combinations with barium aluminum borosilicate glass and ytterbium fluoride). A detailed composition analysis was reported in Table 1. This compositional diversity gave a good opportunity to examine how inorganic and organic phases appear in the reflectance region. The choice of measuring total hemispherical reflectance, which combines both specular and diffuse contributions, proved particularly effective for these opaque and highly scattering materials [21,22]. Figure 6a shows the reflectance spectra of the investigated composites. Although the overall spectral shapes appear broadly similar, distinct absorption features can be recognized in specific spectral regions, and their relative intensities are closely related to the type and fraction of fillers present in the different formulations.

Three distinct regions are observed in the spectra, providing complementary information on the polymer matrix and inorganic fillers composition and properties. At shorter wavelengths (below ∼400 nm), all the samples exhibit intense absorption. Two main bands are observed around 230 nm and 270 nm and are attributed to oxygen-to-network-cation charge-transfer transitions associated with borate (B–O) and silicate (Si–O–Si) structural units present in the borosilicate glass fillers. Similar absorption features have been previously reported for SiO_2_–B_2_O_3_-based glass systems and associated with the electronic structure of borate and silicate network units within the glass matrix [23,24]. The bands are more intense for the barium aluminum borosilicate glass-filled composites than for the system that comprises only silica (SOFT ENA). This illustrates the considerably different sensitivity of UV spectroscopy with respect to filler type and composition, establishing a consistent ‘fingerprint’ of the inorganic phase. The spectra show little absorption in the visible range (400–800 nm). The optical properties of the composites are dominated by scattering that is determined strongly by the size and dispersion of inorganic particles. Among the factors that affect diffuse reflection, filler fraction is a major element and is clinically relevant because it sways translucency, brightness, and overall esthetic effect of a restoration. To obtain natural optical properties, the balance between particle scattering and the transparency of the polymeric matrix should be appropriate. In the near-infrared region between 900 and 1500 nm, the spectra offer complementary information that enables inorganic and organic contributions to be simultaneously determined. VENUS, TETRIC and SIMPLY SHADE composites, including ytterbium fluoride, show a well-defined absorbance band between 900 and 1050 Nm. This band is attributed to the f–f electronic transition bands of Yb^3+^ ions, being indicative evidence of its presence [25,26,27,28]. The lack of this feature in ytterbium fluoride-free materials verifies that spectral features are correlated directly to the filler composition. Furthermore, the organic material contained in the composites—methacrylate polymers—provides specific vibrational signatures in this region. The second band range from 1200 to 1400 nm is dominated by C–H stretching vibration second harmonic mode related to methyl and carbon chain groups, revealing a clear methacrylate-resin fingerprint [29]. In Figure 6b, the ATR-FTIR spectra for the analyzed samples are reported. The ATR-FTIR spectra of all investigated resins exhibit the characteristic features of methacrylate-based polymer networks, including the ester carbonyl stretching band at ∼1715–1730 cm^−1^ and the methacrylate C=C stretching vibration around 1635–1640 cm^−1^, whose very low intensity is consistent with the cured nature of the materials [30,31]. Aromatic peaks should appear around 3100–3000 cm^−1^; however, they are not visible because the spectra are dominated by filler and methacrylate matrix contributions, and aromatic signals are weak or overlapped. Aliphatic C–H stretching modes are observed in the 2850–2960 cm^−1^ range, albeit with limited intensity. The dominant spectral contribution for all samples appears in the 1200–800 cm^−1^ region, where strong and broad absorptions are assigned to Si–O–Si stretching modes of silica- and silicate-based fillers, partially overlapping with C–O–C vibrations of the organic matrix. No pronounced broad O–H stretching band is observed in the 3200–3600 cm^−1^ region, indicating negligible moisture or bound hydroxyl groups, in agreement with the minimal low-temperature mass loss observed in the low-temperature range of TGA profiles. While the overall spectral profiles are similar across the investigated resins, SOFT ENA shows more pronounced features in the fingerprint region, suggesting differences in filler composition and/or polymer blend, consistent with the trends observed in UV, SEM–EDX, and thermogravimetric analyses. Owing to the coexistence of multiple methacrylate polymers with similar functional groups and the strong contribution of inorganic fillers, FTIR does not allow an unambiguous identification of individual polymeric components but provides qualitative confirmation of the composite nature of the resins.

### 3.4. Rheological Characterization

**Figure 7:**
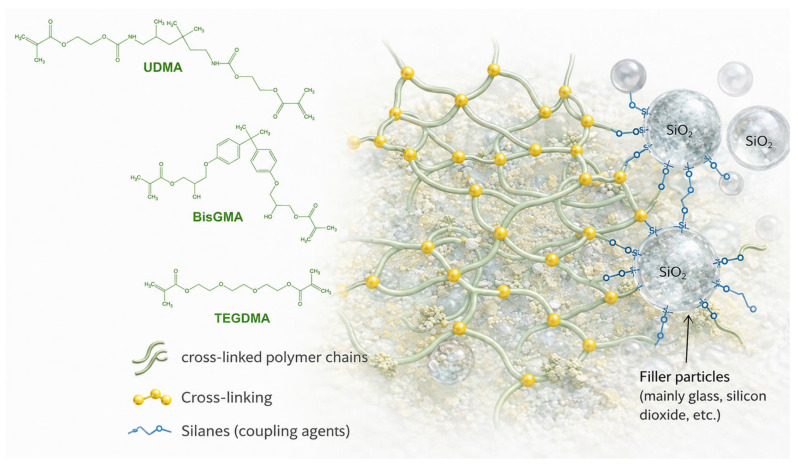
Schematic representation of the resulting composite architecture, illustrating the entangled polymer chains embedded within a densely filled inorganic matrix, with filler particles (e.g., silica) chemically coupled to the resin via silane agents acting as interfacial linkers [32,33].

The structural and compositional complexity revealed by the previous characterization techniques indicates that the investigated resins are heterogeneous composite systems, in which multiple polymeric components coexist with a significant fraction of inorganic fillers. Such complexity is expected to play a key role in governing the macroscopic mechanical response of the materials. Overall, the mechanical performance of these composite resins is expected to arise from a combination of polymer–polymer interactions, crosslinking among the different chains, interactions between the polymeric matrix and the inorganic fillers, and filler–filler interactions. A schematic representation of the resulting composite architecture, illustrating the entangled polymer chains embedded within a densely filled inorganic matrix, is shown in Figure 7, providing a conceptual framework for interpreting the experimental results. In this context, rheological characterization provides a suitable tool to probe how these microstructural features translate into viscoelastic behavior, flow properties, and processability. Therefore, the rheological behavior of the resins, both before and after curing, is discussed in detail in the following sections.

#### 3.4.1. Viscosity of Uncured Resins

**Figure 8:**
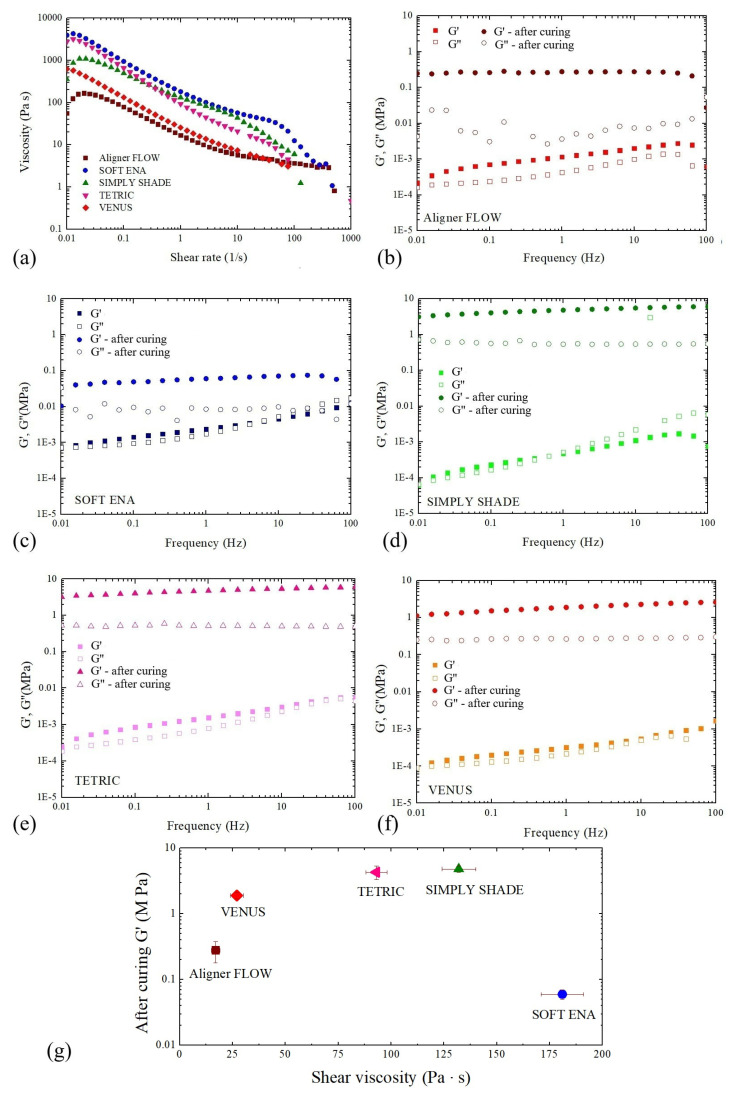
(**a**) Double logarithmic Flow curve (Viscosity vs. Shear rate) for all the tested resins; Frequency sweep results for uncured and cured resins: (**b**) Aligner FLOW, (**c**) SOFT ENA, (**d**) SIMPLY SHADE, (**e**) TETRIC and (**f**) VENUS; (**g**) Comparison of shear viscosity (Pa·s) and post-curing storage modulus G′ (MPa) for the investigated dental resins.

Starting with viscosity, all resins exhibited what is known as shear-thinning behavior (Figure 8a). As the shear rate increased, their resistance to flow decreased, which is exactly what is expected in a dental material. In practice, this means they can be manipulated easily under the pressure of a syringe or applicator yet remain relatively stable once placed. Among the five, SOFT ENA stood out as the most viscous material across the range of shear rates. Its thick, paste-like consistency implies that it may offer excellent handling during sculpting, particularly where precise anatomical shaping is important. However, that same viscosity might also make it more difficult to dispense into narrow attachment molds or fine geometries. At the opposite end of the spectrum, Aligner FLOW was notably the least viscous. It flowed easily, suggesting excellent adaptability to small features and fast placement. This ease of use may be especially beneficial in clinical scenarios where speed and precision are important. Yet, such low viscosity could come with trade-offs, specifically, the risk that the material might migrate or slump slightly before curing, especially if not handled carefully. The other resins, SIMPLY SHADE, TETRIC, and VENUS, show intermediate viscosity values (Figure 8a). Their viscosities suggest a balanced formulation that provides both reasonable flow under pressure and sufficient resistance to unintended movement. This makes them likely candidates for general-purpose use, where both control and adaptability are needed in equal measure.

#### 3.4.2. Viscoelastic Behavior Before and After Curing

Moving from flow behavior to mechanical performance, the frequency sweep tests revealed some unexpected but important insights. Before curing, all five resins already demonstrated a degree of elastic behavior (Figure 8b–f). In fact, in most of the frequency range tested, the storage modulus (G′), which reflects the material’s ability to store energy elastically, was greater than or equal to the loss modulus (G″), which captures viscous energy dissipation. This is somewhat unusual, as one typically expects liquid resins to behave more like viscous fluids than soft solids [34]. Yet in this case, even the uncured materials showed a dominant elastic response. Clinically, this could be quite useful: a resin that holds its shape well before curing is less likely to sag or displace, allowing more accurate placement of attachments. After curing, the transformation was pronounced. As expected, all resins exhibited a strong increase in both G′ and G″, with G′ becoming significantly higher. This shift confirms that polymer crosslinking had taken place effectively, and that the resins had transitioned from viscoelastic fluids to elastic solids [35,36]. Aligner FLOW, despite its ease of application, displayed a moderate mechanical transformation (Figure 8b). After curing, its G′ increased as expected, but not to the same extent as the other materials. This suggests a somewhat softer final structure, which may be advantageous in areas where excessive rigidity is not desirable, or where patient comfort is a key concern. However, it may be less appropriate for attachments subject to high mechanical stress. SOFT ENA presented a curious contrast (Figure 8c). Despite its high viscosity in the uncured state, something that usually signals a more crosslink-dense formulation, it resulted in the softest final material after curing. One possible explanation, supported by the low inorganic residue observed in TGA, is that the organic-rich nature of SOFT ENA limits crosslink density and/or generates weaker interfacial zones, ultimately leading to reduced hardness after curing. Moreover, it suggests that SOFT ENA’s formulation prioritizes pre-cure handling and sculptability over post-cure rigidity. This could be beneficial in specific clinical applications, such as attachments that do not need to bear high loads but require exact anatomical shaping. Among the tested resins, SIMPLY SHADE and TETRIC demonstrated the most striking change (Figure 8d,e). Their storage modulus after curing reached levels several orders of magnitude higher than in the uncured state, resulting in highly rigid, well-structured materials. These two resins appear to form dense, stiff networks upon curing, making them excellent candidates for attachment applications that demand mechanical strength and dimensional stability under aligner forces.

VENUS shows a similar behavior (Figure 8f). Its stiffness after curing was substantial, but not as high as SIMPLY SHADE or TETRIC. Nevertheless, it maintained a strong elastic character and would likely perform well in most clinical scenarios. What makes VENUS particularly attractive is its balanced nature; it flows well enough before curing to allow easy placement yet cures into a sufficiently robust structure for durable function. The comparative plot in Figure 8g summarizes the rheological and mechanical behavior of the investigated dental resins by correlating shear viscosity with post-curing storage modulus (G′). Overall, higher viscosity does not necessarily correspond to higher stiffness. TETRIC and SIMPLY SHADE combine high viscosity (≈90–135 Pa·s) with the highest post-curing G′ (~5 MPa), indicating rigid cured networks but thicker, less flowable formulations. VENUS and Aligner FLOW are low-viscosity materials (≈15–25 Pa·s) with moderate G′, suggesting easier processability but lower stiffness than the most rigid composites. In contrast, SOFT ENA shows the highest viscosity (≈180 Pa·s) yet the lowest G′ (∼0.05–0.1 MPa). Overall, the graph highlights the trade-off between processability (viscosity) and final mechanical rigidity among the analyzed formulations.

### 3.5. Flat Indentation of the Cured Resin

**Figure 9:**
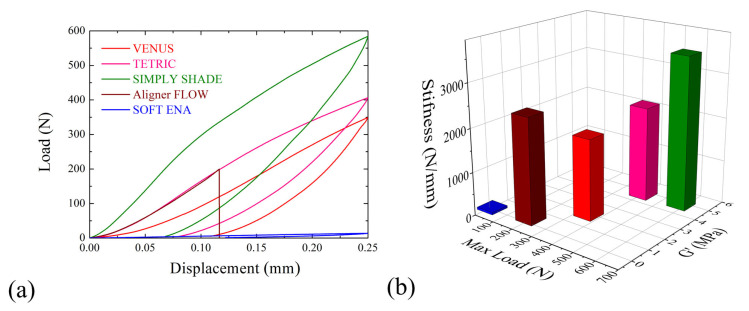
(**a**) Loading and unloading curves of the flat indentation tests; (**b**) Comparison of post-curing storage modulus G′ (MPa), Maximum load (N) and Stiffness (N/mm) for the investigated dental resins.

The resistance of the composites was evaluated via flat indentation tests. Figure 9a illustrates the load–displacement curves.

The flat-indentation results reveal clear differences in the mechanical response of the tested orthodontic resins, particularly in terms of stiffness, load-bearing capacity, and deformation behavior [37]. Among the materials, SIMPLY SHADE exhibits the highest stiffness, as indicated by its steep initial slope and its ability to sustain the greatest load at a given displacement, reaching approximately 590 N at 0.25 mm (Table 3). This suggests a highly rigid structure with strong resistance to indentation. In contrast, SOFT ENA shows a markedly lower load response across the entire displacement range, confirming its highly compliant, elastomer-like behavior and limited structural resistance. VENUS and TETRIC demonstrate intermediate behavior, combining moderate stiffness with pronounced nonlinearity, which is indicative of elastic–plastic deformation and possible strain hardening under increasing load. The presence of hysteresis in their loading–unloading paths further suggests viscoelastic energy dissipation, a property that may be advantageous for absorbing stresses in clinical applications. Aligner FLOW displays relatively high initial stiffness but is characterized by a sudden drop in load around 0.1 mm displacement due to fracture during testing, pointing to limited deformation tolerance. The elastic modulus, calculated from the slope of the linear region of the curve in the stress–strain graph, confirms the trends discussed. The mechanical results confirm a clear difference in stiffness and load-bearing capacity of the investigated resins. SIMPLY SHADE showed the highest performance, with a maximum load of 584 N, contact stiffness of 3504 N/mm, and an elastic modulus approaching 950 MPa, indicating a highly rigid network. TETRIC follows with slightly lower but still elevated values (E ≈ 610 MPa), while VENUS shows moderate mechanical properties (E ≈ 544 MPa), consistent with a less rigid structure. Aligner FLOW, despite its relatively low viscosity, demonstrates mechanical properties comparable to TETRIC (E ≈ 613 MPa), suggesting that adequate crosslinking density can be achieved even in more flowable systems. In contrast, SOFT ENA exhibits drastically lower mechanical performance (E ≈ 18.5 MPa), confirming its soft, compliant nature and indicating a significantly lower crosslinking density.

Figure 9b shows the comparison between the storage modulus G′ derived from the rheological characterizations and the Maximum Load and stiffness derived from flat indentation tests. The 3D graph highlights an overall agreement between storage modulus (G′) and stiffness among the five orthodontic resins: samples with higher G′ generally exhibit higher stiffness. The SIMPLY SHADE shows the best overall mechanical performance, with the highest G′, maximum load, and stiffness, whereas the SOFT ENA shows the lowest values for all parameters. The Aligner FLOW resin represents the main exception, displaying relatively high stiffness despite low G′ and limited maximum load, which indicates a brittle response with early failure under loading. TETRIC and VENUS samples fall in an intermediate range, with increasing G′ associated with increasing stiffness.

Overall, the data highlights a spectrum of mechanical performance, from soft and highly deformable to stiff and load-resistant materials, which is critical for tailoring resin selection to specific orthodontic requirements. Mechanical analysis emphasizes the trade-off between processability and mechanical performance. These findings underline the importance of formulation design in achieving a balance between clinical handling and structural performance in dental applications.

## 4. Discussion

### 4.1. Microstructural and Compositional Implications for Orthodontic Attachments

In this study, five flowable composite resins were evaluated to determine their suitability for the fabrication of orthodontic attachments used in clear aligner therapy. Unlike conventional restorative applications, attachment fabrication relies on a process in which, after tooth surface etching, primer application and light cure, the composite is injected in a single step into the aligner template, which acts as a negative mold. In this context, the material must simultaneously ensure precise flow in microgeometries, efficient polymerization, and sufficient mechanical stability to maintain the programmed shape under cyclic loading.

The microstructural characterization provided insight into the internal organization of the investigated composites. SEM/EDX analyses indicated that all materials exhibited a generally homogeneous distribution of inorganic fillers within the polymer matrix. Such a microstructural arrangement may contribute to a more uniform stress distribution and reduce the likelihood of localized stress concentrations that could act as initiation sites for microcracks under repeated insertion and removal cycles [38].

Considering that orthodontic attachments function as the mechanical interface between tooth enamel and the aligner, variations in structural integrity or surface morphology may influence the consistency of force transmission and aligner tracking, although this relationship cannot be directly quantified within the present experimental design [39].

Among the investigated materials, Aligner FLOW and TETRIC showed a particularly uniform filler dispersion. While no direct clinical correlation can be established, this feature may be associated with improved dimensional stability and a more consistent mechanical response under cyclic loading. Compositional analysis further highlighted the role of the inorganic phase. The presence of barium glass fillers and ytterbium fluoride contributes to radiopacity and may also influence mechanical properties such as stiffness and resistance to deformation. In general, a higher filler content combined with effective matrix–filler interactions is expected to reduce deformation under load, potentially improving the transfer of forces, particularly during complex movements such as rotations, extrusions, and torque control [7].

Thermogravimetric analysis provided complementary information regarding the inorganic fraction of the composites. Materials such as Aligner FLOW, SIMPLY SHADE, TETRIC, and VENUS exhibited higher residual masses after thermal degradation, suggesting a greater filler content and, consequently, a potential increase in stiffness and wear resistance. These characteristics may contribute to maintaining attachment geometry under repeated mechanical loading, although long-term clinical performance cannot be inferred directly from these findings.

Conversely, SOFT ENA showed a lower residual mass, indicating a relatively higher organic content, and post-curing low stiffness, as demonstrated both from rheological tests and via flat indentation measurements [37].

Overall, the combined SEM/EDX and TGA results suggest that filler distribution, matrix–filler interactions, and inorganic content are key factors influencing the mechanical response of these materials. Although direct clinical implications cannot be established, these findings support the importance of considering microstructural and compositional characteristics, in addition to handling properties, when selecting materials for orthodontic attachment fabrication [37,40].

### 4.2. Optical and Rheological Implications for Clinical Performance

In addition to microstructural and compositional aspects, the behavior of orthodontic attachments is influenced by optical and rheological properties, which affect both esthetic integration and fabrication accuracy. UV–Vis spectroscopy revealed differences among the investigated composites across the UV, visible, and near-infrared regions, reflecting variations in filler composition and polymer matrix formulation. In the UV region (<400 nm), intense absorption bands associated with B–O and Si–O transitions confirmed the presence of borosilicate and silica-based fillers, with more pronounced signals observed in materials containing barium–aluminum–borosilicate glass compared to silica-only systems such as SOFT ENA.

In the visible range (400–800 nm), absorption was limited, and optical behavior was mainly governed by scattering phenomena related to filler size, dispersion, and volume fraction. Materials such as SOFT ENA and VENUS showed higher reflectance, suggesting a potentially improved optical blending with the surrounding tooth structure under the aligner, although this aspect was not directly evaluated clinically [40]. In the near-infrared region (900–1500 nm), composites containing ytterbium fluoride (VENUS, TETRIC, and SIMPLY SHADE) exhibited characteristic bands attributable to Yb^3+^ ions, confirming the presence of radiopaque fillers.

ATR-FTIR analysis confirmed the formation of polymerized methacrylate networks in all materials, with dominant Si–O–Si bands indicating the contribution of silica-based fillers. The absence of significant O–H absorption suggests limited moisture uptake, which may reduce susceptibility to hydrolytic degradation over time.

Rheological measurements showed that all materials exhibited shear-thinning behavior prior to polymerization, facilitating injection into the aligner template and potentially improving reproduction of the planned attachment geometry. This behavior is consistent with previous studies reporting improved flowability, reduced overflow, and enhanced geometric accuracy for flowable composites compared with more viscous formulations [41,42,43].

Following light curing, all composites exhibited a marked increase in stiffness, indicating effective network formation. The flat-ended indentation tests further supported these observations, showing differences in surface mechanical response among the materials. In particular, SIMPLY SHADE exhibited the highest stiffness, followed by TETRIC and VENUS, while Aligner FLOW showed intermediate values, and SOFT ENA displayed the lowest stiffness, confirming its softer mechanical behavior.

Overall, the combined spectroscopic, rheological, thermogravimetric, and mechanical findings highlight that these materials exhibit distinct but overlapping property profiles. Rather than identifying a single optimal composite, the results suggest that differences in filler composition, optical response, and post-curing stiffness may influence material behavior under clinical conditions. The performance of orthodontic attachments is therefore likely dependent on the interaction between material properties, attachment design, and aligner characteristics, rather than on a single defining parameter [37,40,44,45].

## 5. Conclusions

This study comparatively evaluated five commercially available flowable composites used for clear aligner attachments through integrated rheological, mechanical, thermal, optical, and morphological analyses.

The main findings can be summarized as follows:

- Post-curing mechanical properties varied significantly, with storage modulus spanning nearly two orders of magnitude (~0.06–5 MPa).
- Elastic modulus ranged from ~20 MPa to ~1000 MPa, highlighting substantial differences in stiffness among materials.
- SEM/EDX and TGA analyses showed overall consistency with manufacturer specifications, while revealing subtle variations in filler content and heterogeneous filler distribution.
- UV–Vis–NIR spectroscopy provided distinct spectral fingerprints, reflecting differences in filler composition and polymer matrix structure.

Stiffer, highly filled materials such as SIMPLY SHADE and TETRIC may be preferable for attachments requiring high dimensional stability and force transmission, while lower-viscosity materials such as Aligner FLOW and VENUS may be advantageous where template filling and handling are prioritized. SOFT ENA may be useful for adaptation and sculptability but appears less suitable for highly loaded attachments. These findings provide a physicochemical framework to support informed material selection in clear aligner therapy.

Building on these results, future developments may benefit from extended experimental approaches, including in situ photorheological characterization, long-term aging and cyclic loading assessments, as well as clinical performance tracking, to further strengthen the correlation between laboratory properties and real-world clinical behavior.

## Figures and Tables

**Table 1:** Composition of the five commercial dental resins as reported by the manufacturer.

Sample Name	Matrix Composition	Filler Composition
Aligner FLOW LC VOCO GmbH, Anton-Flettner-Str. 1–3, 27472 Cuxhaven, Germany)	HEDMA/Bis GMA/TEGDMA/BisEMA	Barium aluminum borosilicate glass, silica, pigments
SIMPLY SHADE Kerr Corporation, 200 S. Kraemer Blvd., Brea, CA 92821, USA (Part of Envista Holdings Corporation)	BisEMA/BisGMA/TEGDMA	Barium aluminum borosilicate glass, silica, ytterbium fluoride, pigments
SOFT ENA Flow Micerium S.p.A., Via Guglielmo Marconi, 83, 16036 Avegno (GE), Italy	UDMA/HEDMA	Silica, pigments
TETRIC EvoFlow Ivoclar Vivadent AG, S.r.l., Via Isonzo 69, 40033 Casalecchio di Reno (BO), Italy	DMA	Barium aluminum borosilicate glass, silica, ytterbium fluoride, titanium dioxide, pigments
VENUS Bulk Flow One Kulzer GmbH, Leipziger Str. 2, 63450 Hanau, Germany	EGBADMA/UDMA	Barium aluminum borosilicate glass, silica, ytterbium fluoride, titanium dioxide, pigments

**Table 2:** Curing procedure parameters.

Sample	Irradiation Time (s) t	Light Intensity (mW/cm^2^) I	Total Energy Density (J/cm^2^) E = t × I
VENUS	20	1000	20
SIMPLY SHADE	20	1000	20
SOFT ENA	20	1000	20
TETRIC	10	1000	10
Aligner FLOW	20	1000	20

**Table 3:** Features derived from flat-indentation tests.

Sample	Max Load at 0.25 mm (N)	Contact Stiffness (N/mm)	Elastic Modulus (MPa)
VENUS	350 ± 10	1900 ± 80	550 ± 50
TETRIC	410 ± 20	2200 ± 100	610 ± 60
SIMPLY SHADE	590 ± 20	3500 ± 200	950 ± 80
SOFT ENA	12 ± 2	54 ± 6	19 ± 2
Aligner FLOW	270 ± 10	2400 ± 80	610 ± 70

## Data Availability

The data supporting the findings of this study are available from the corresponding author upon reasonable request. The authors are open to sharing the research data generated and analyzed during this study.

## Supplementary Materials

The following supporting information can be downloaded at: https://www.mdpi.com/article/10.3390/polym18111308/s1, Figure S1. Representative clinical examples of composite attachments used in CAT. The attachments, fabricated from dental resin composites, are strategically placed on tooth surfaces to enhance aligner retention and improve the transmission of orthodontic forces. Black arrows: retention attachments, Blu arrows: rotation attachments. Green arrows: rotation-extrusion attachments, Red arrows: occlusal beveled horizontal attachment. Pink arrows: rectangular-vertical attachment; Figure S2: SEM micrographs of the different samples, acquired at 50 kX magnification and an accelerating voltage of 3 keV; Figure S3: EDX spectra and elemental maps for Aligner Flow cured resin; Figure S4: EDX spectra and elemental maps for SOFT ENA cured resin; Figure S5: EDX spectra and elemental maps for SIMPLY SHADE cured resin; Figure S6: EDX spectra and elemental maps for TETRIC cured resin; Figure S7: EDX spectra and elemental maps for VENUS cured resin. References [46,47,48,49,50,51] are cited in Supplementary Materials.

## Abbreviations

The following abbreviations are used in this manuscript:

Bis-EMA	Bisphenol A diglycidyl methacrylate ethoxylated
Bis-GMA	Bisphenol A-glycidyl methacrylate
CAD	Computer-aided design
CAM	Computer-aided manufacturing
CAT	Clear aligner therapy
DMA	Dimetacrylate
DTG	Derivative thermogravimetric
DUDMA	Diurethane dimethacrylate
EDX	Energy-Dispersive X-ray Spectroscopy
EGBADMA	Ethylene glycol bisphenol A dimethacrylate
EVA	Ethylene vinyl acetate
HEDMA	Hydroxyethyldimethacrylate
LVR	Linear viscoelastic region
SEM	Scanning electron microscopy
TEGDMA	Triethylene Glycol Dimethacrylate
TGA	Thermogravimetric analyses
UDMA	Urethane dimethacrylate
