# Supplementary material for: Rheological and Structural Evaluation of Dental Flowable Composites for Optimized Performance in Transparent Aligner Systems
Source: Polymers (Basel). 2026 May 26;18(11):1308. doi: 10.3390/polym18111308 (PMC13259519; doi:10.3390/polym18111308)
Supplement: Supplementary file 1 [file polymers-18-01308-s001.zip › polymers-4221415-supplementary.pdf]

polymers

MDPI

# SUPPLEMENTARY INFORMATION

# Rheological and structural Evaluation of Dental Flowable Composites for Optimized Performance in Transparent Aligner Systems

**Elena Palmieri [1], Maria Elena Cataldi [2], Loredana Cerroni [2], Luca Montaina [1], Matteo Bonomo [3,4], Gaetana Petrone [1,5], Denise Bellisario [6], Leonardo Mattiello [3,4], Guido Pasquantonio [2], Andrea Liscio [1], Francesco Maita [1,*], Luca Maiolo[1] and Roberta Condò [2,*]**

[1] Institute for microelectronics and microsystems, National Research Council, CNR-IMM, Via del Fosso del Cavaliere, 100, 00133, Rome, Italy;
[2] Department of Clinical Sciences and Translational Medicine, University of Rome "Tor Vergata", Via Montpellier, 1, 00133, Rome, Italy;
[3] Department of Basic and Applied Science for Engineering, SBAI Sapienza University of Rome, Via del Castro Laurenziano, 7, 00161, Rome, Italy;
[4] Research Center for Nanotechnologies Applied to Engineering, CNIS Sapienza University of Rome, Piazzale Aldo Moro, 5, 00161, Rome, Italy;
[5] Department of Electrical and Energy Engineering, DIEE Sapienza University of Rome, Via Eudossiana, 18, 00184, Rome, Italy;
[6] Department of Industrial Engineering, University of Rome "Tor Vergata", Via del Politecnico, 1, 00133, Rome, Italy.
* Correspondence: francesco.maita@cnr.it; roberta.condo@uniroma2.it

**Citation:** To be added by editorial staff during production.

Academic Editor: Firstname Lastname

Received: date
Revised: date
Accepted: date
Published: date

**Copyright:** © 2026 by the authors. Submitted for possible open access publication under the terms and conditions of the Creative Commons Attribution (CC BY) license (https://creativecommons.org/licenses/by/4.0/).

## Rationale for Attachments in Aligner Therapy

Clear aligner systems achieve orthodontic tooth movement through staged activation of thermoplastic aligners, which apply controlled forces on the dentition. However, due to their elastic nature and limited thickness, aligners alone may fail to achieve precise control over certain tooth movements, such as root torque, bodily translation, or vertical displacements. The aligner tends to slip or deform on the smooth tooth surface, compromising force direction and magnitude [1,2]. Attachments, typically made of composite resins, are bonded directly to the tooth enamel to function as geometric anchor points (Figure S1). Their role is multifold: they increase the contact surface and friction with the aligner, enhance the retention of the aligner itself, and provide mechanical features that redirect forces to better control the movement of individual teeth. For example, beveled or rectangular attachments can deliver optimized couples for rotation or root movement, while ellipsoid or rectangular attachments can improve extrusion or intrusion [3].

In clinical practice, attachments are often obtained using flowable composite resins, chosen primarily for their good adaptability to guide aligners or templates, but also for their ease of application and moderate mechanical strength. However, the chemical composition of these materials, including the types of monomers, filler content, and photopolymerization system, represent variables that can directly influence their clinical behavior, dimensional stability, degree of adaptation, and ability to withstand the stresses induced by the aligners [4]. The ideal attachment should maintain its shape under repeated insertion and removal of the aligner, resist wear and stress fatigue, and avoid detachment over the entire orthodontic treatment (6-12 months). It should also present good aesthetics, ensuring patients do not perceive it as a hindrance [5]. At the same time, the thermoplastic polymers used to produce aligners (mainly PETG, PU, and modified PMMA, etc.) also possess specific mechanical and rheological properties [6].

 https://doi.org/10.3390/polym18111308

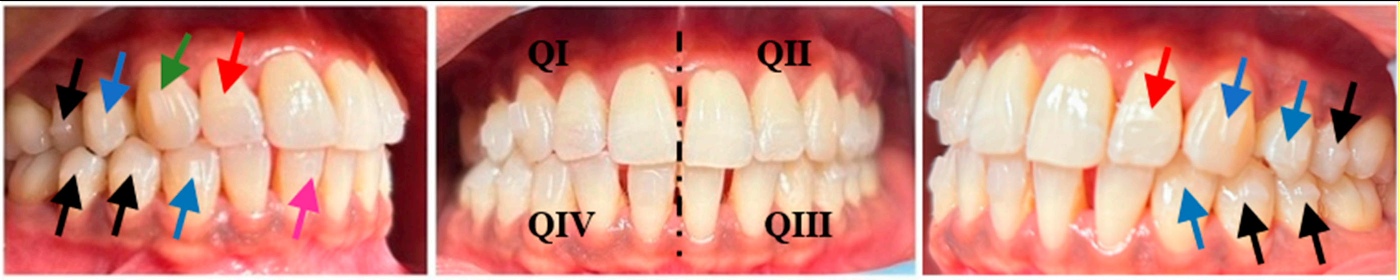


**Figure S1.** Representative clinical examples of composite attachments used in CAT. The attachments, fabricated from dental resin composites, are strategically placed on tooth surfaces to enhance aligner retention and improve the transmission of orthodontic forces. Black arrows: retention attachments, Blu arrows: rotation attachments. Green arrows: rotation-extrusion attachments, Red arrows: occlusal beveled horizontal attachment. Pink arrows: rectangular-vertical attachment.

More specifically in the case study presented: QUADRANT I, Upper right second premolar (15): retention attachment; Upper right first premolar (14): rotation attachment; Upper right canine (13): rotation-extrusion attachment; Upper right lateral incisor (12): occlusal beveled horizontal attachment; QUADRANT II, Upper left second premolar (25): retention attachment; Upper left first premolar (24): rotation attachment; Upper left canine (23): rotation attachment; Upper left lateral incisor (22): occlusal beveled horizontal attachment; QUADRANT III, Lower left second premolar (35): retention attachment; Lower left first premolar (34): retention attachment; Lower left canine (33): rotation attachment; QUADRANT IV, Lower right second premolar (45): retention attachment; Lower right first premolar (44): retention attachment; Lower right canine (43): rotation attachment; Lower right central incisor (41): rectangular-vertical attachment.

It is logical to assume that, to ensure good mechanical-functional interaction between the attachment and the aligner, it is therefore desirable that the viscoelastic and mechanical characteristics of the flowable composite resin of the attachment be compatible with those of the main polymer of the orthodontic aligner. In the context of increasing digitalization of orthodontics, optimizing the attachment/composite-aligner/polymer material interface represents a strategic objective to ensure the efficacy, durability, and predictability of orthodontic treatment [7]. A clinical aspect closely linked to these dynamics is the phenomenon of tracking, that is, the aligner's ability to faithfully follow the prescribed treatment plan at each therapeutic stage. Inadequate tracking occurs when the aligner no longer perfectly fits the teeth or the attachment, or when tooth movements are incomplete or inefficient, compromising treatment progression and forcing to the creation of new sets of aligners or refinements, with a consequent lengthening of treatment times and further environmental impact. Therefore, the composite resin used to realize it must combine excellent bonding capability, adequate rigidity, viscoelastic properties compatible with the repeated mechanical cycling of aligner seating, and a color/translucency matching the surrounding enamel. Despite the widespread use of flowable composite resins for attachments realization, clinicians often base material selection predominantly on general properties rather than orthodontic application-specific evidence, risking mechanical mismatches that might compromise aligner tracking and treatment predictability [8].

**Morphological and compositional characterization of cured resins**

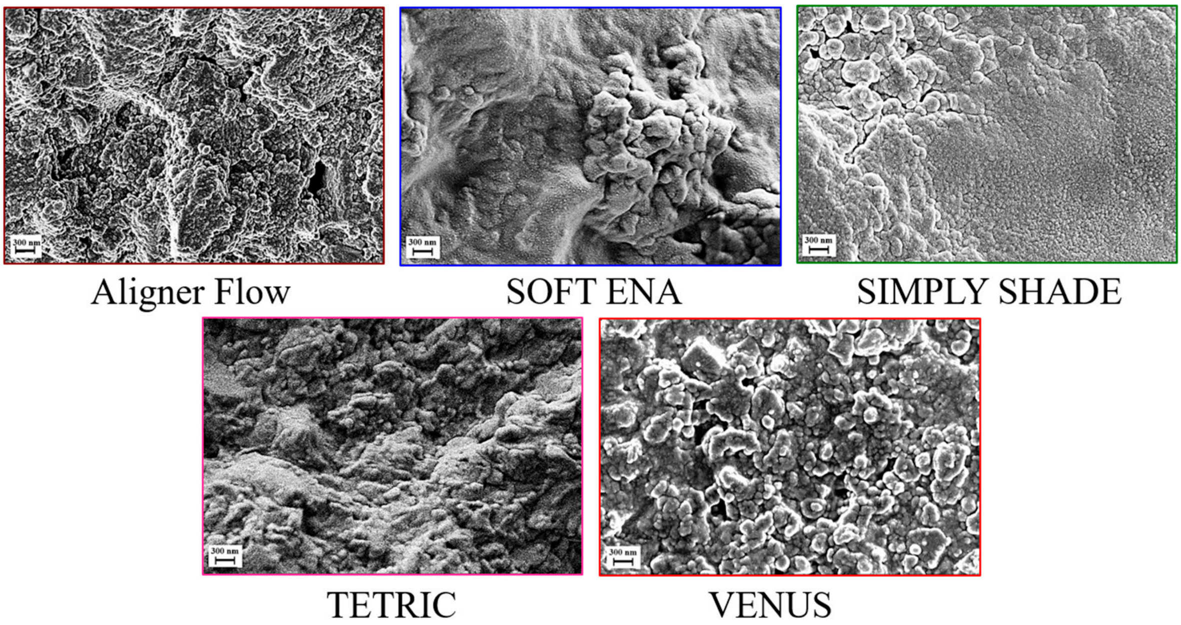


**Figure S2:** SEM micrographs of the different samples, acquired at 50 kX magnification and an accelerating voltage of 3 keV

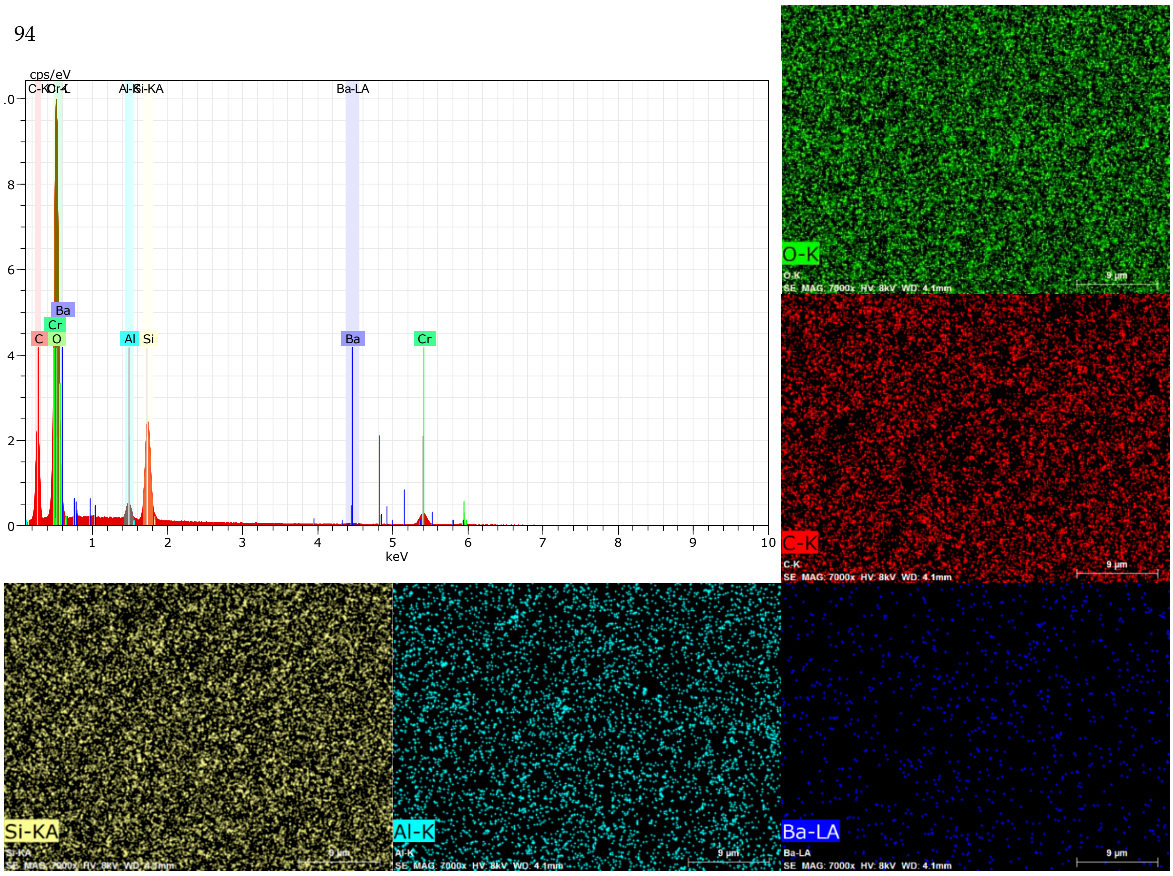


**Figure S3:** EDX spectra and elemental maps for Aligner Flow cured resin.

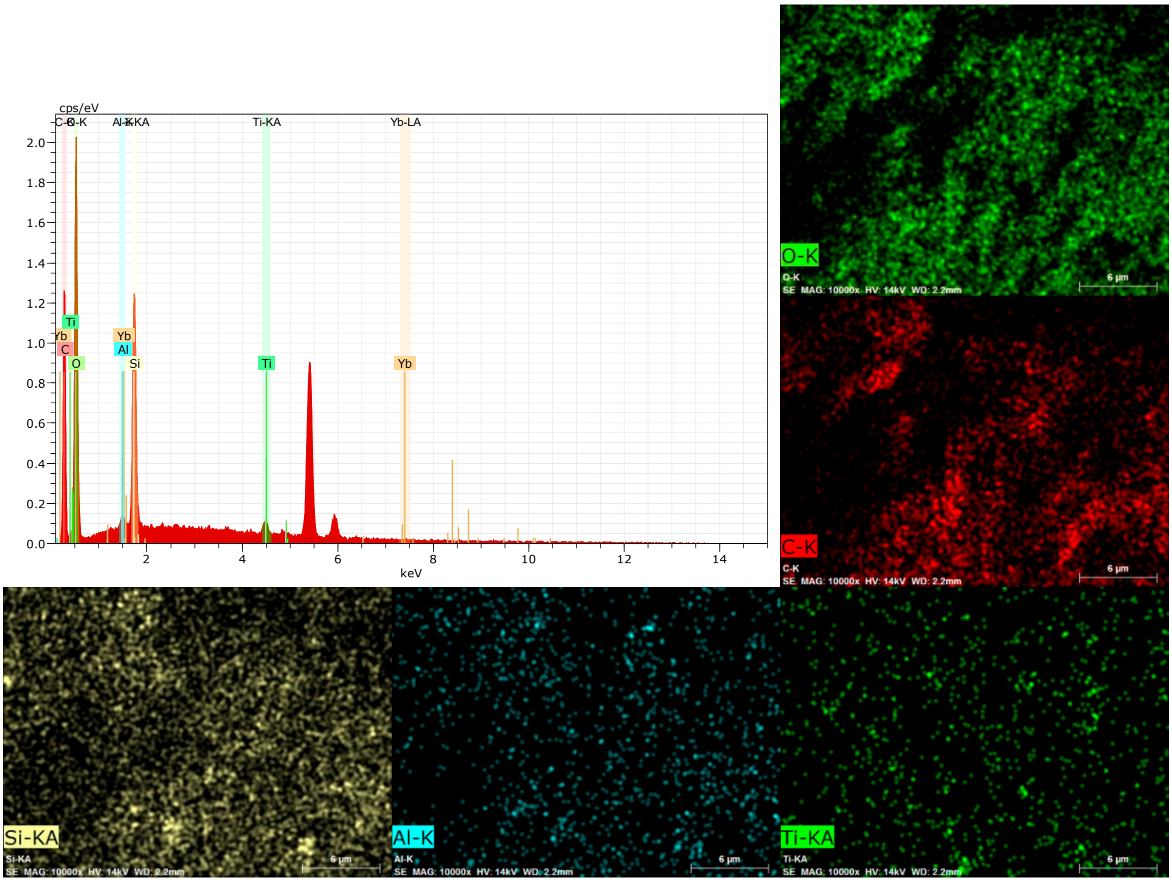


**Figure S4:** EDX spectra and elemental maps for SOFT ENA cured resin.

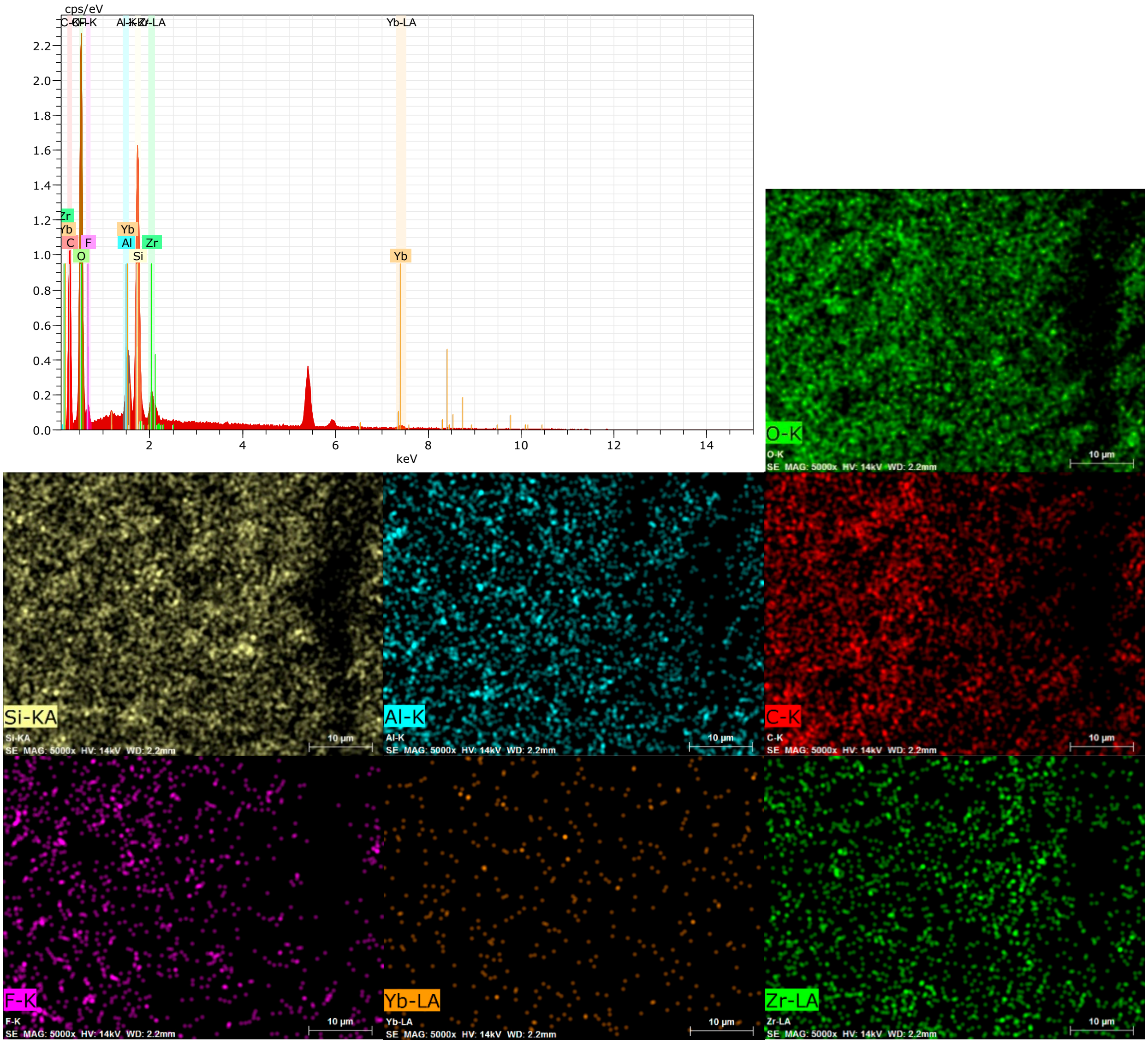


**Figure S5**: EDX spectra and elemental maps for SIMPLY SHADE cured resin.

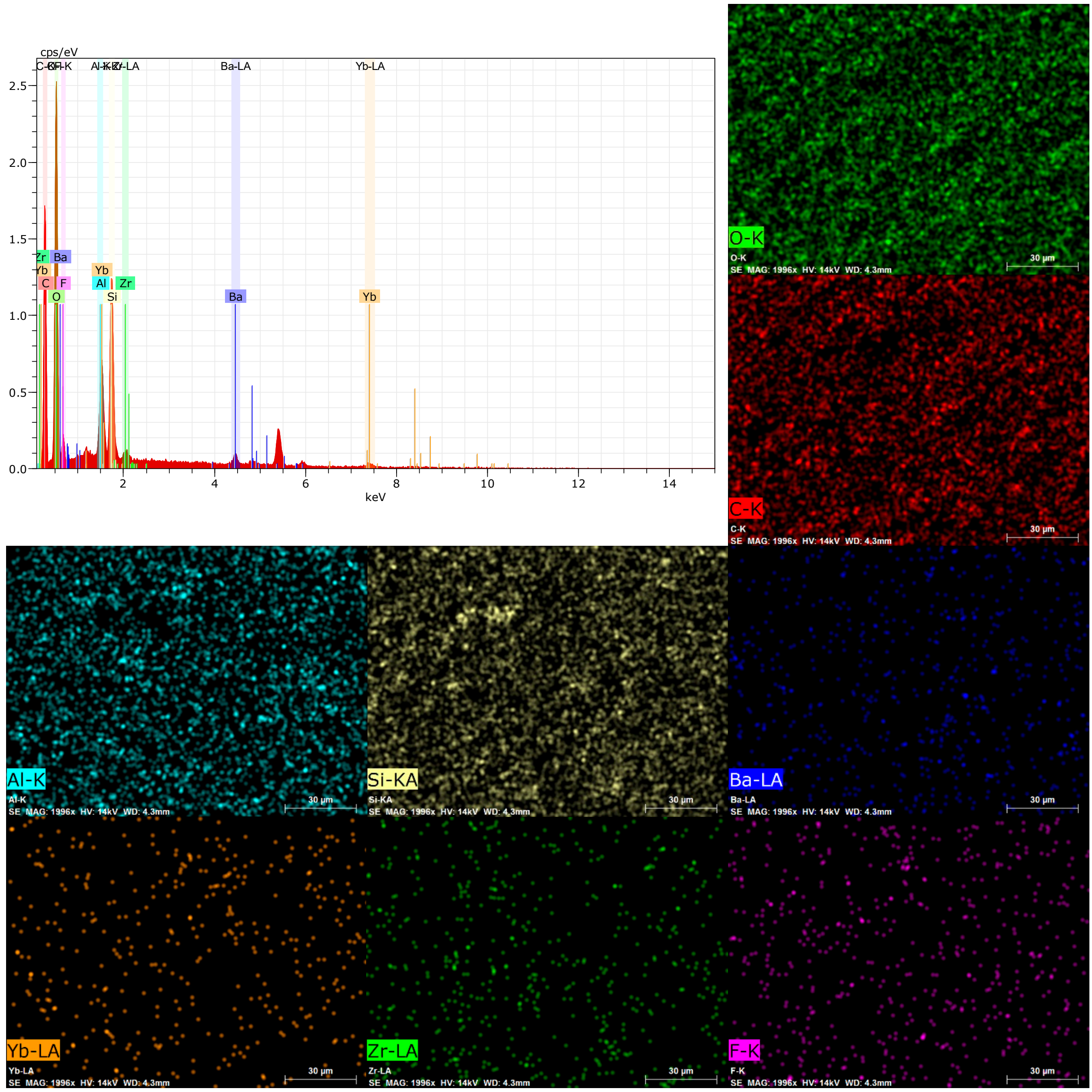

**Figure S6**: EDX spectra and elemental maps for TETRIC cured resin.

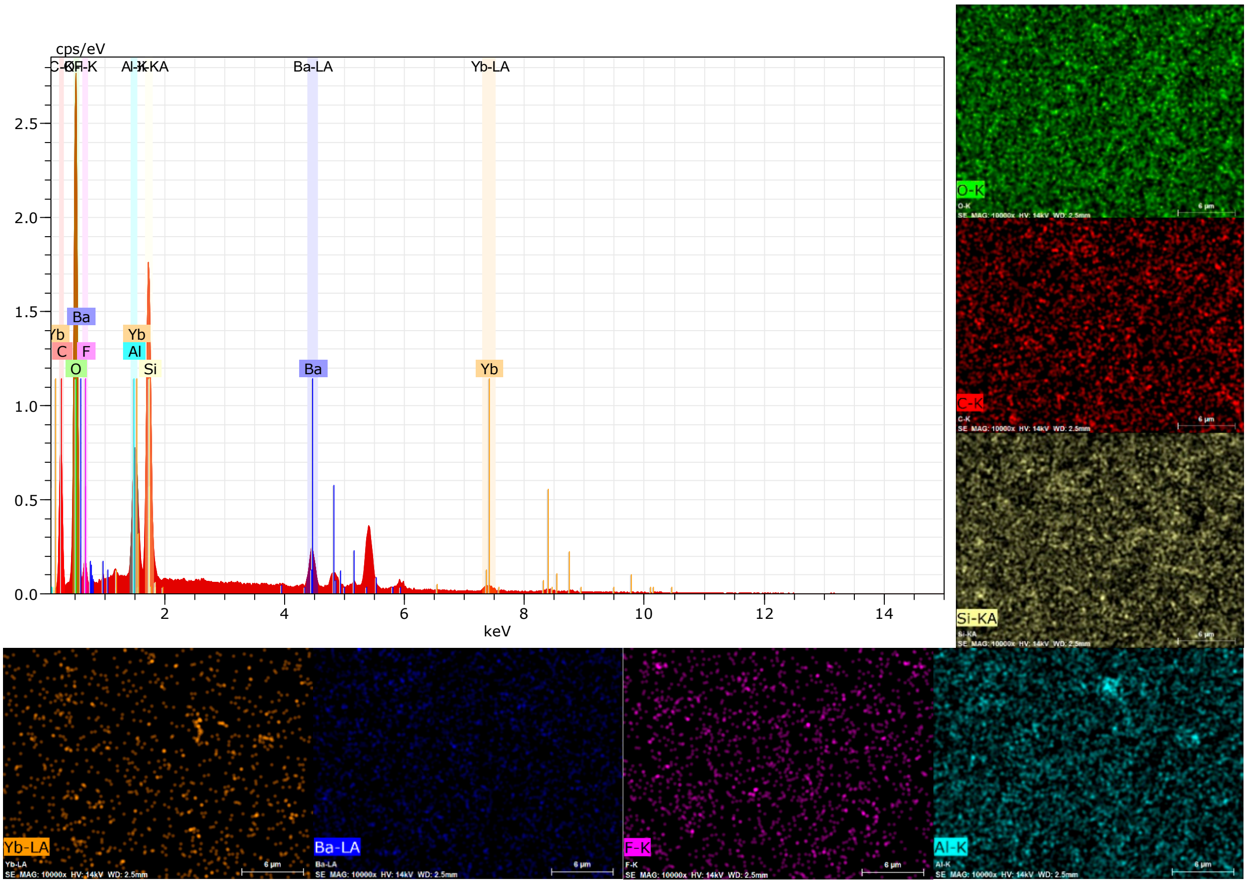


**Figure S7:** EDX spectra and elemental maps for VENUS cured resin.

## Reference


1. Mantovani, E.; Castroflorio, E.; Rossini, G.; Garino, F.; Cugliari, G.; Deregibus, A.; Castroflorio, T. Scanning Electron Microscopy Analysis of Aligner Fitting on Anchorage Attachments. *J. Orofac. Orthop.* **2019**, *80*, 79–87, doi:10.1007/S00056-018-00167-1.
2. Ravera, S.; Castroflorio, T.; Garino, F.; Daher, S.; Cugliari, G.; Deregibus, A. Maxillary Molar Distalization with Aligners in Adult Patients: A Multicenter Retrospective Study. *Prog. Orthod.* **2016**, *17*, doi:10.1186/S40510-016-0126-0.
3. Yangın, A.; Camcı, H.; Soybelli, M. Clear Aligner Attachments: A Comprehensive Review. *Turk. J. Orthod.* **2025**, *38*, 177–189, doi:10.4274/TURKJORTHOD.2025.2025.7.
4. Chen, W.; Qian, L.; Qian, Y.; Zhang, Z.; Wen, X. Comparative Study of Three Composite Materials in Bonding Attachments for Clear Aligners. *Orthod. Craniofac. Res.* **2021**, *24*, 520–527, doi:10.1111/OCR.12465.
5. Nucera, R.; Dolci, C.; Bellocchio, A.M.; Costa, S.; Barbera, S.; Rustico, L.; Farronato, M.; Militi, A.; Portelli, M. Effects of Composite Attachments on Orthodontic Clear Aligners Therapy: A Systematic Review. *Materials (Basel)* **2022**, *15*, doi:10.3390/MA15020533.
6. Gold, B.P.; Siva, S.; Duraisamy, S.; Idaayath, A.; Kannan, R. Properties of Orthodontic Clear Aligner Materials - A Review. *J. Evol. Med. Dent. Sci.* **2021**, *10*, 3288–3294, doi:10.14260/JEMDS/2021/668.
7. Iliadi, A.; Zervou, S.K.; Koletsi, D.; Schätzle, M.; Hiskia, A.; Eliades, T.; Eliades, G. Surface Alterations and Compound Release from Aligner Attachments in Vitro. *Eur. J. Orthod.* **2024**, *46*, doi:10.1093/EJO/CJAE026.
8. Yildiz, H.; Dedeoglu, M. Reliability of Different Composite Materials in Aligner Treatments: A Comprehensıve in Vitro Study. Prog. Orthod. 2025, 26, doi:10.1186/S40510-025-00594-Y.
